# Supplementary material for: Acute Toxicity of Carbon Nanotubes, Carbon Nanodots, and Cell-Penetrating Peptides to Freshwater Cyanobacteria
Source: Toxins (Basel). 2025 Apr 1;17(4):172. doi: 10.3390/toxins17040172 (PMC12031272; doi:10.3390/toxins17040172)
Supplement: Supplementary file 1 [file toxins-17-00172-s001.zip › toxins-3484123-supplementary.pdf]

Supplementary Materials

Acute Toxicity of Carbon Nanotubes, Carbon Nanodots, and Cell-Penetrating Peptides to Freshwater Cyanobacteria

Anna K. Antrim, Ilana N. Tseytlin, Emily G. Cooley, P. U. Ashvin Iresh Fernando, Natalie D. Barker, Erik M. Alberts, Johanna Jernberg, Gilbert K. Kosgei and Ping Gong

Part 1. This part presents the photos of control and exposed cyanobacterial cultures. Nine cyanobacterial strains were used in this study, including two benthic strains, i.e., *Microcoleus autumnale* CAWBG635 ATX and *Lyngbya* sp. CCAP 1446/10 (see **Figure S1** for evidence).

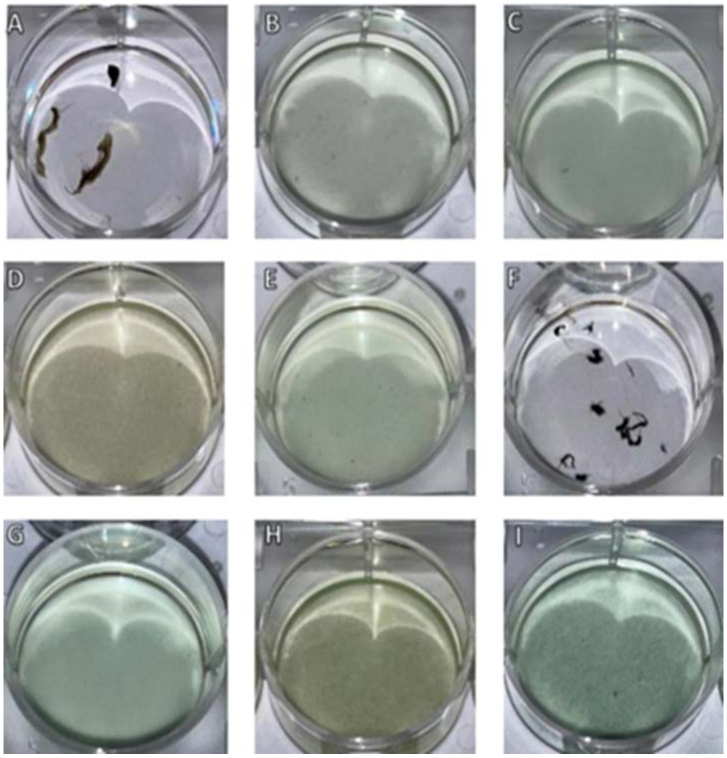

Figure S1. Photos of untreated laboratory cultures maintained in multi-well plates to demonstrate the benthic nature of *Microcoleus* sp. (A) and *Lyngbya* sp. (F) strains. (A) *Microcoleus autumnale* CAWBG635 ATX; (B) *Microcystis aeruginosa* UTEX 2385; (C*) Microcystis aeruginosa* UTEX 2386; (D) *Planktothrix agardhii* SB 1810; (E) *Microcystis aeruginosa* LE3; (F*) Lyngbya* sp. CCAP 1446/10; (G) *Synechocystis* sp. PCC 6803; (H) *Aphanizomenon* sp. NZ; and (I) *Anabaena cylindrica* PCC 7122.

The following are images of 9 cyanobacterial cultures (5 mL per sample) taken 48 hours after treatment with two batches of carbon nanodots (CND-G and CND-C/M), one fraction (A14) of another CND-G batch (CND-G-A14), one batch of single-walled carbon nanotubes (SWCNT), or the cell penetrating peptide (CPP) γ-zein-CADY at two concentrations (high and low with high = 2 × low) in comparison with the control group. All exposures were performed in 25-mL flasks. The samples of SWCNT treatments and controls were transferred to 6-well plates for post-treatment imaging. The CND-G, CND-C/M and CPP treatments shared the same control group.

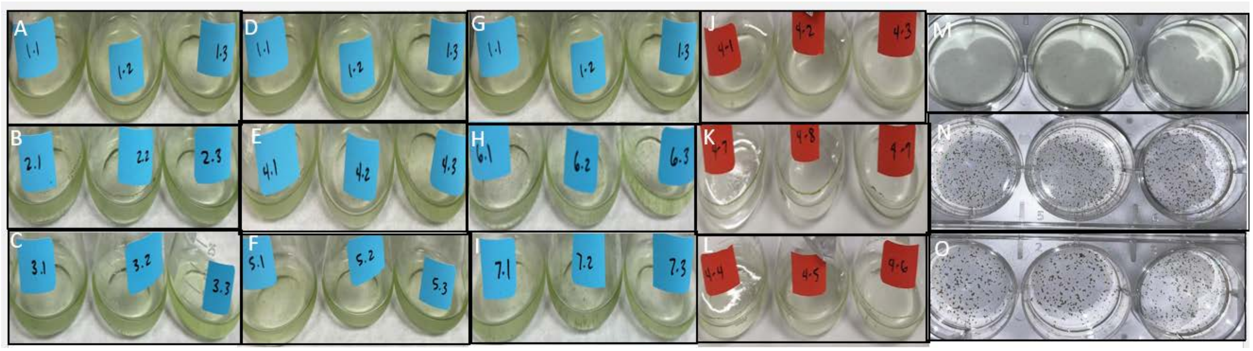

Figure S2. Photos of *Microcystis aeruginosa* UTEX 2385. (A) Control; (B) CND-G_high; (C) CND-G_low; (D) Control (same as A); (E) CND-C/M_high; (F) CND-C/M_low; (G) Control (same as A); (H) CPP_high; (I) CPP_low; (J) Control; (K) CND-G-A14_high; (L) CND-G-A14_low; (M) Control; (N) SWCNT_high (O) SWCNT_low.

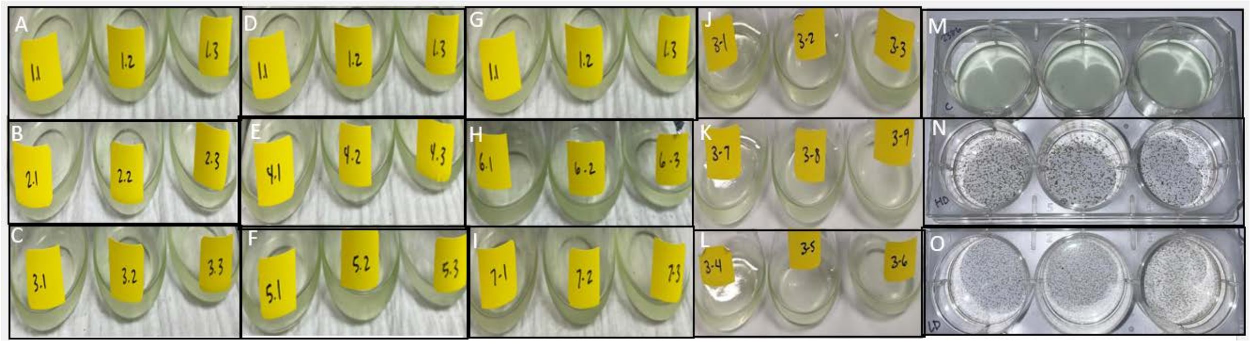

Figure S3. Photos of *Microcystis aeruginosa* UTEX 2386. (A) Control; (B) CND-G_high; (C) CND-G_low; (D) Control (same as A); (E) CND-C/M_high; (F) CND-C/M_low; (G) Control (same as A); (H) CPP_high; (I) CPP_low; (J) Control; (K) CND-G-A14_high; (L) CND-G-A14_low; (M) Control; (N) SWCNT_high (O) SWCNT_low.

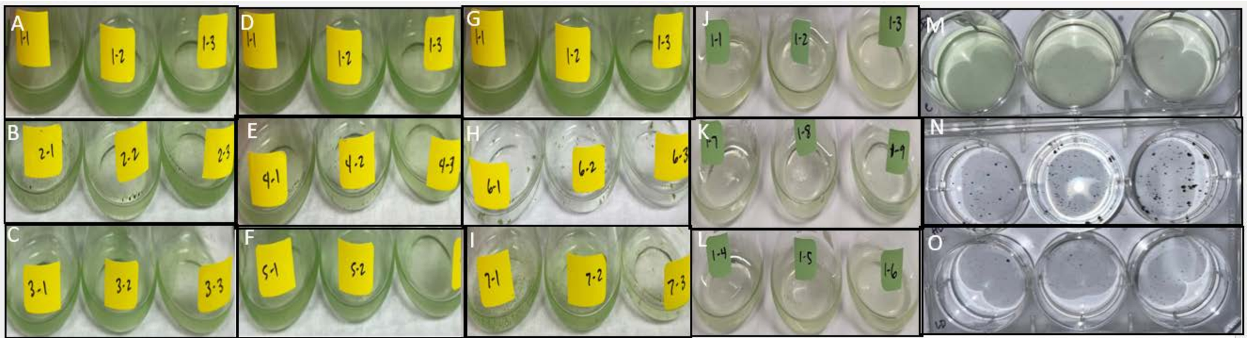

Figure S4. Photos of *Microcystis aeruginosa* LE3. (A) Control; (B) CND-G_high; (C) CND-G_low; (D) Control (same as A); (E) CND-C/M_high; (F) CND-C/M_low; (G) Control (same as A); (H) CPP_high; (I) CPP_low; (J) Control; (K) CND-G-A14_high; (L) CND-G-A14_low; (M) Control; (N) SWCNT_high (O) SWCNT_low.

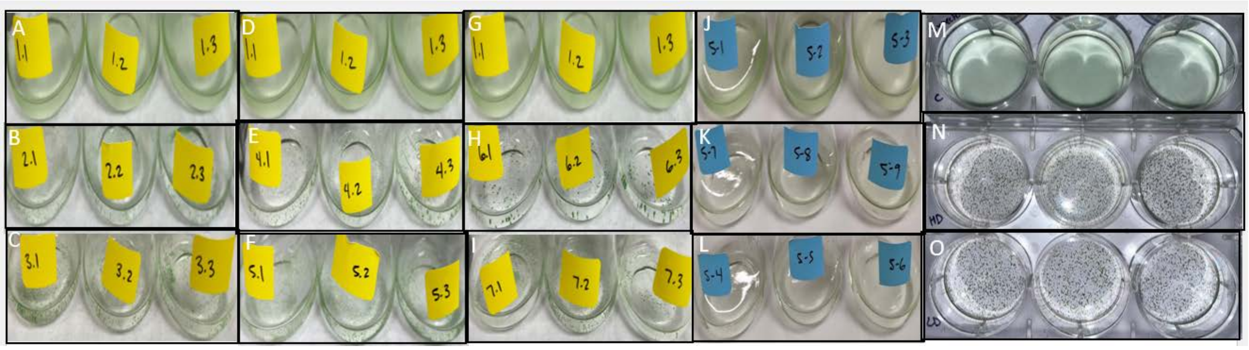

Figure S5. Photos of *Synechocystis* sp. PCC 6803. (A) Control; (B) CND-G_high; (C) CND-G_low; (D) Control (same as A); (E) CND-C/M_high; (F) CND-C/M_low; (G) Control (same as A); (H) CPP_high; (I) CPP_low; (J) Control; (K) CND-G-A14_high; (L) CND-G-A14_low; (M) Control; (N) SWCNT_high (O) SWCNT_low.

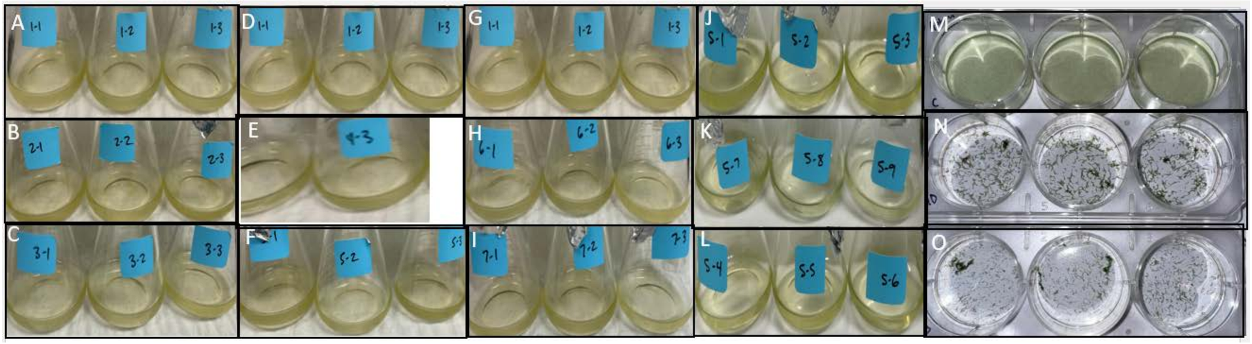

Figure S6. Photos of *Aphanizomenon* sp. NZ. (A) Control; (B) CND-G_high; (C) CND-G_low; (D) Control (same as A); (E) CND-C/M_high; (F) CND-C/M_low; (G) Control (same as A); (H) CPP_high; (I) CPP_low; (J) Control; (K) CND-G-A14_high; (L) CND-G-A14_low; (M) Control; (N) SWCNT_high (O) SWCNT_low.

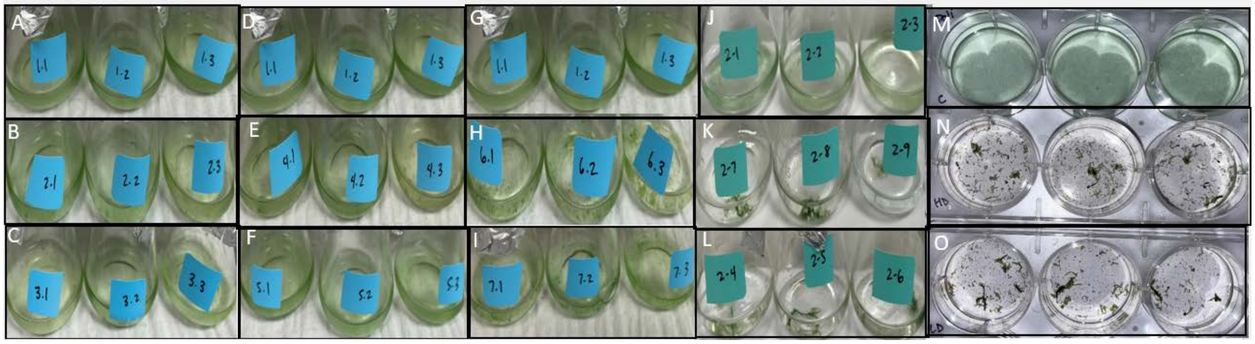

Figure S7. Photos of *Anabaena cylindrica PCC 7122*. (A) Control; (B) CND-G_high; (C) CND-G_low; (D) Control (same as A); (E) CND-C/M_high; (F) CND-C/M_low; (G) Control (same as A); (H) CPP_high; (I) CPP_low; (J) Control; (K) CND-G-A14_high; (L) CND-G-A14_low; (M) Control; (N) SWCNT_high (O) SWCNT_low.

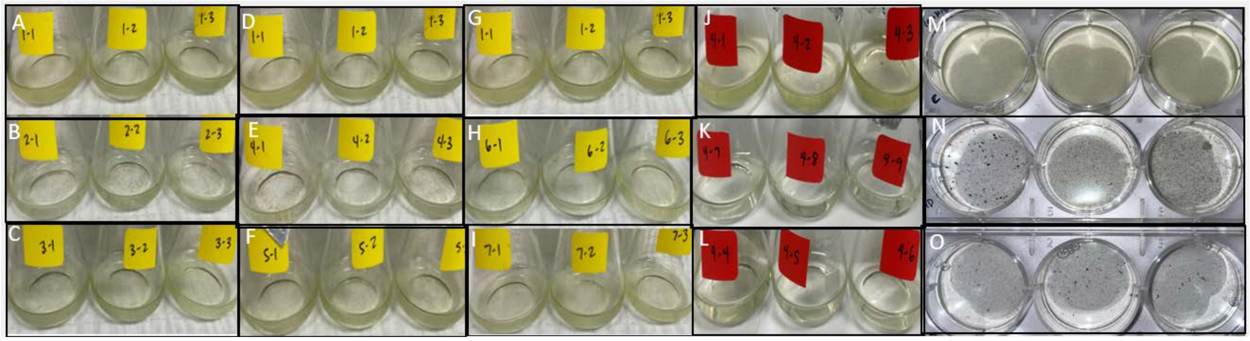

Figure S8. Photos of *Planktothrix agardhii* SB 1810. (A) Control; (B) CND-G_high; (C) CND-G_low; (D) Control (same as A); (E) CND-C/M_high; (F) CND-C/M_low; (G) Control (same as A); (H) CPP_high; (I) CPP_low; (J) Control; (K) CND-G-A14_high; (L) CND-G-A14_low; (M) Control; (N) SWCNT_high (O) SWCNT_low.

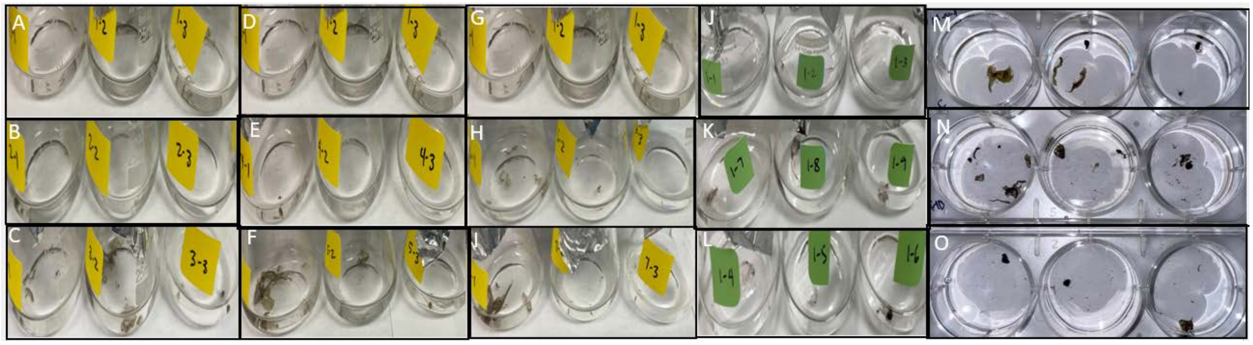

Figure S9. Photos of *Microcoleus autumnale* CAWBG635 ATX. (A) Control; (B) CND-G_high; (C) CND-G_low; (D) Control (same as A); (E) CND-C/M_high; (F) CND-C/M_low; (G) Control (same as A); (H) CPP_high; (I) CPP_low; (J) Control; (K) CND-G-A14_high; (L) CND-G-A14_low; (M) Control; (N) SWCNT_high (O) SWCNT_low.

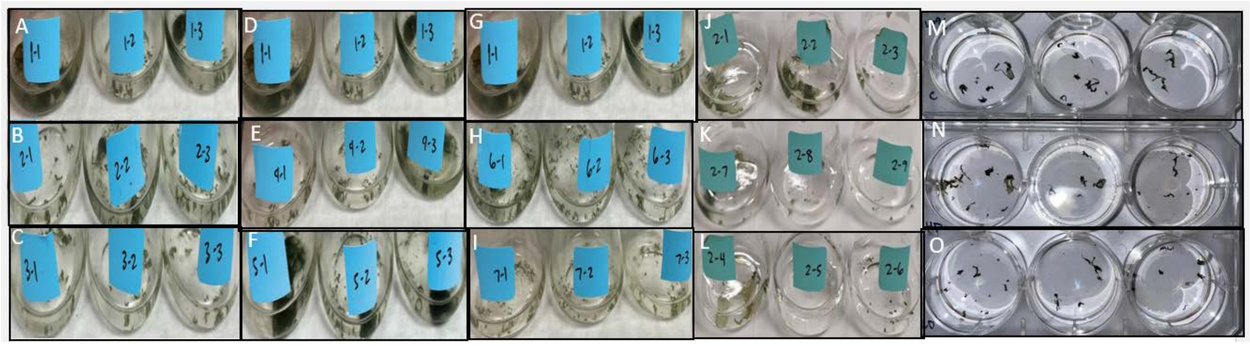

Figure S10. Photos of *Lyngbya* sp. CCAP 1446/10. (A) Control; (B) CND-G_high; (C) CND-G_low; (D) Control (same as A); (E) CND-C/M_high; (F) CND-C/M_low; (G) Control (same as A); (H) CPP_high; (I) CPP_low; (J) Control; (K) CND-G-A14_high; (L) CND-G-A14_low; (M) Control; (N) SWCNT_high (O) SWCNT_low.

Part 2. CND synthesis, purification, fractionation, and characterization

Reference: Schwarz, S.; Hendrix, B.; Hoffer, P.; Sanders, R.; Zheng, W. Carbon Dots for Efficient Small Interfering RNA Delivery and Gene Silencing in Plants. Plant Physiology 2020, 184(2), 647–657.

2.1 Fraction A14 from CNDs synthesized from glucose and 10-kDa bPEI (CND-G-A14)

CNDs were first synthesized from glucose and 10-kDa bPEI (branched polyethylenimine) as described in the **Methods and Materials** section. Then the CNDs were lyophilized and used to prepare a stock solution of 40 mg CND/3 mL, which was diluted to generate a standard curve. An UV/Vis detector was used to measure the absorbance. Absorption maxima was found to be 362 nm (**Figure S11a**). Absorbance peak values at the wavelength 362 nm were used to construct the standard curve (**Figure S11b**).

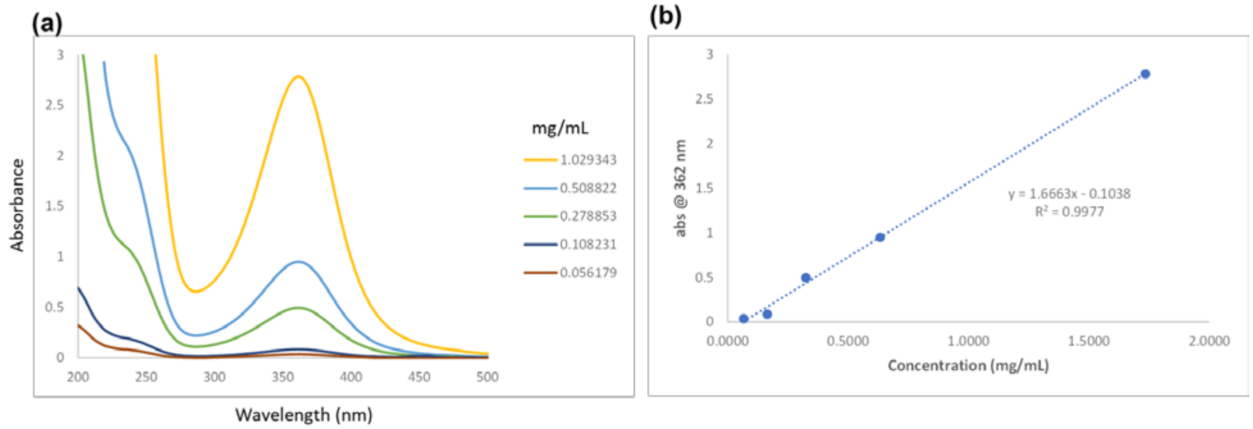

Figure S11. (a) Absorption curves for synthesized CND solutions (0.056 ~ 1.029 mg/mL) serially diluted from the stock solution (40 mg/3 mL) showing peak absorbance at 362 nm, and (b) a linear relationship curve between CND concentration and absorbance at 362 nm constructed using data derived from the absorption curves in (a).

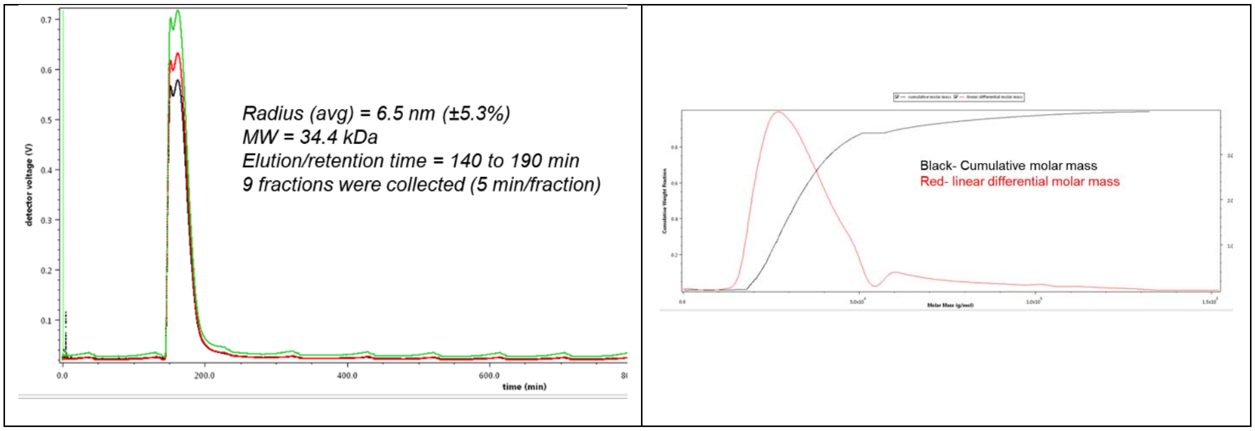

Figure S12. (a) Chromatogram for the synthesized CNDs, and (b) graph for cumulative weight fraction vs. molar mass.

A Bio-Rad NGC Quest 10 Chromatography system equipped with A Bio-Rad Econo column (2.5 × 20 cm) packed with Sephadex G-50 resin and a MALS/RI (Multiangle light scattering /Refractive Index) detector was used to purify and fractionate the synthesized CNDs. The column was eluted at 1 mL/min with 50 mM NaCl while elution of the CNDs was monitored at 360 nm (**Figure S12**). Fractions of 5 mL each were collected starting after 30 mL. Nine of the collected fractions (A9 to A17) were quantified for their CND concentrations (**Figure S13**) and characterized for particle size and intensity distribution (**Figures S14** & **S15**). As measured using Dynamic Light Scattering (DLS) on a Malvern Zetasizer Nano ZS, the size distribution of each fraction was characterized by two peaks, one for small particles (e.g., 6 nm for A14) and the other for large particles (e.g., 810 nm for A14) (**Figure S14**).

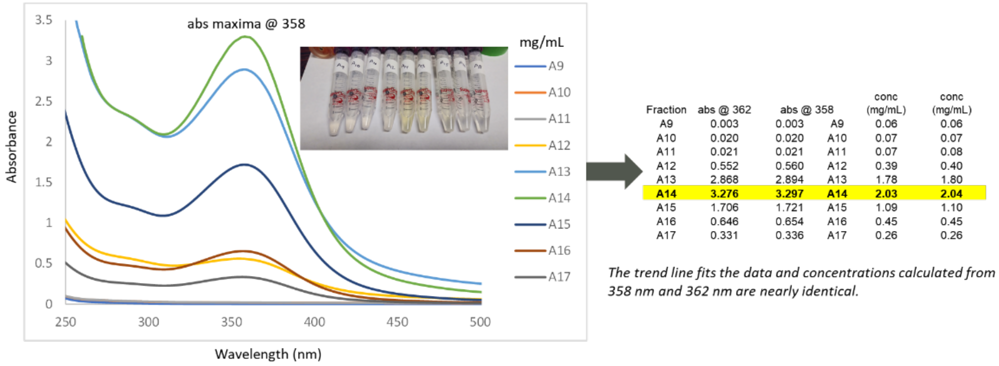

Fraction	abs @ 362	abs @ 358		conc (mg/mL)	conc (mg/mL)
A9	0.003	0.003	A9	0.06	0.06
A10	0.020	0.020	A10	0.07	0.07
A11	0.021	0.021	A11	0.07	0.08
A12	0.552	0.560	A12	0.39	0.40
A13	2.868	2.894	A13	1.78	1.80
**A14**	**3.276**	**3.297**	**A14**	**2.03**	**2.04**
A15	1.706	1.721	A15	1.09	1.10
A16	0.646	0.654	A16	0.45	0.45
A17	0.331	0.336	A17	0.26	0.26

The trend line fits the data and concentrations calculated from 358 nm and 362 nm are nearly identical.

Figure S13. Absorbance curves for 9 collected fractions and their CND concentrations inferred from the standard curve (see **Figure S11b**)

			Size (d.nm):	% Intensity:	St Dev (d.n...
Z-Average (d.nm):	278.7	Peak 1:	810.8	62.0	135.4
PdI:	0.908	Peak 2:	6.126	38.0	1.047
Intercept:	0.876	Peak 3:	0.000	0.0	0.000
Result quality :	Refer to quality report				

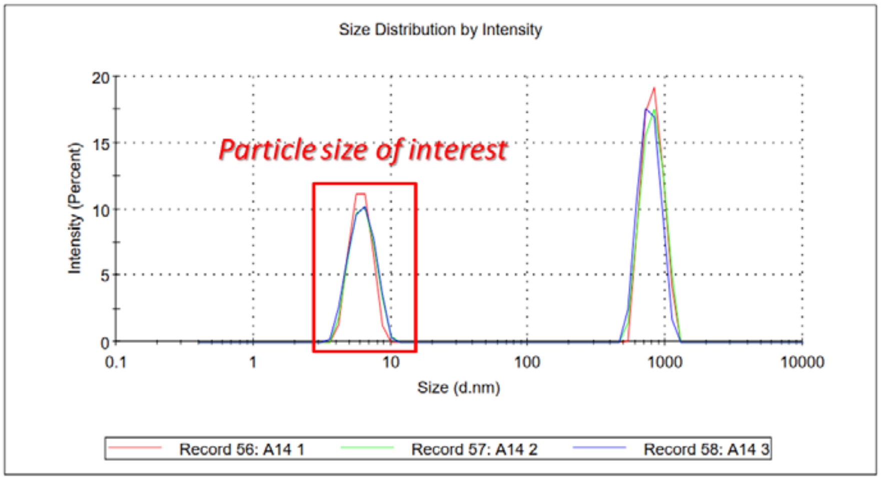

Figure S14. Particle size measurement using Dynamic Light Scattering (DLS) for Fraction A14

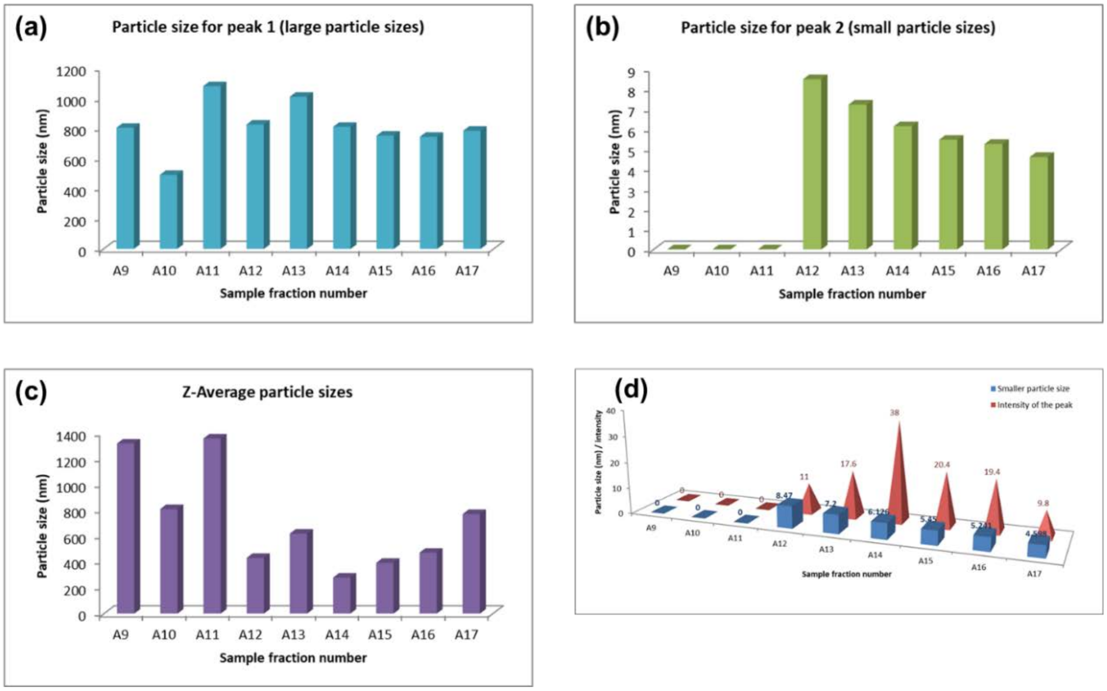

Figure S15. Particle size measurement and intensity distribution of the 9 collected fractions from a PEI-functionalized CND preparation.

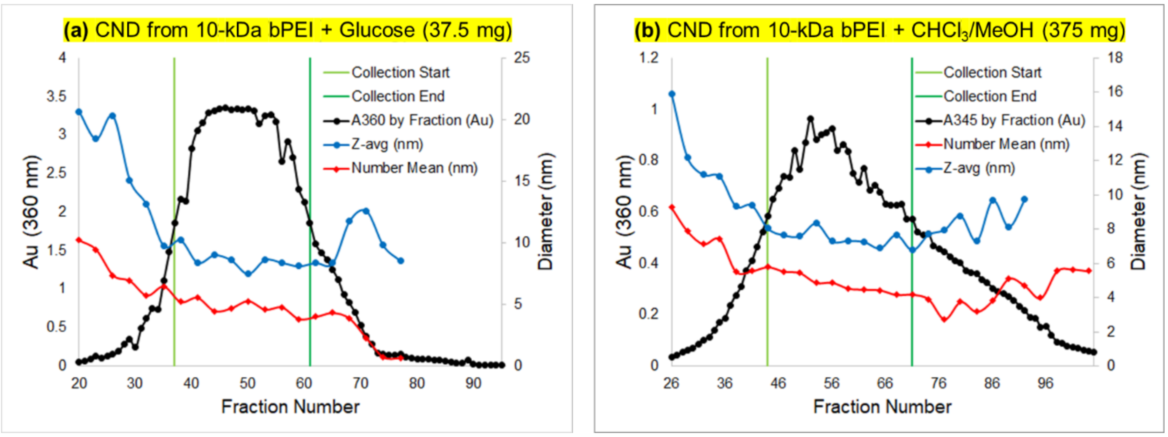

Figure S16. Absorbance at 360 nm and particle size measurement for two CND preparation fractionated and collected using a FPLC (Fast Protein Liquid Chromatography). (a) CND-G synthesized from bPEI and glucose; (b) CND-C/M synthesized from bPEI and chloroform:methanol (4:1)

2.2 Fractionation and characterization of two batches of CNDs (CND-G and CND-C/M)

Two additional batches of CNDs were synthesized using the two methods described in Schwartz et al. (2020) with modification. One batch (CND-G) was synthesized from 10-kDa bPEI and glucose, following the same method as used in 2.1. The other batch (CND-C/M) was prepared from 10-kDa bPEI and $CHCl_3$:MeOH (4:1). A Bio-Rad BioLogic Duo Flow FPLC system equipped with a QuadTec UV/Vis detector and a 2.5 × 20 cm Econo-column (Bio-Rad) filled with Sephadex G-50 superfine (GE Healthcare)

was used to purify and fractionate both batches of CNDs. The column was eluted at 1 mL/min with 50 mM NaCl, and 1 mL/fraction was collected. Elution of the CNDs was monitored at 360 nm for CND-G or 345 nm for CND-C/M. Fractions 37-61 for the CND-G and fractions 44-71 for the CND-C/M were consolidated and characterized using DLS on a Zetasizer (**Figure S16**). The two consolidated CNDs were used for toxicity testing.

Part 3. Preparation and characterization of bPEI-functionalized SWCNT

Reference: Demirer, G.S., Zhang, H., Goh, N.S. et al. Carbon nanotube–mediated DNA delivery without transgene integration in intact plants. Nature Protocol 2019, 14, 2954-2971.

3.1. Preparation of carboxylic acid functionalized single-walled carbon nanotubes 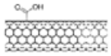 *(SWCNT-COOH) suspension*

Vacuum dried SWCNT-COOH (Sigma-Aldrich; 30 mg) was weighed into a 50-ml conical tube, to which nuclease-free water was added. This solution was bath sonicated for 10 minutes at room temperature (Visible observation: suspension becomes dark black in solution). Solution was then probe-tip sonicated continuously for 30 minutes with a 3.5-mm probe tip, at 30~40% amplitude. No pulses were used. The probe sonication was carried out in an ice bath to avoid over heating on CNT, which may cause the CNT to rupture (**Figure S17**). It is essential that throughout the 30-min sonication the ice bath is well maintained. The above solution was then centrifuged at 18,000g for 1 hour at room temperature. Supernatant was collected, leaving aside the pellet. It is important to collect the supernatant without disturbing the pellet. Absorbance of this supernatant was measured at 632 nm. For the batch of SWCNT-COOH suspension used in the present study, an absorbance value of 0.781 was obtained for the 10 times diluted sample. The Beer-Lambert law was applied to determine the concentration of chemical compounds that absorb light. That is,

$A = \varepsilon \, l \, c$

Where A = absorbance

ε = molar absorptivity (L mol^{-1} cm^{-1})

l = length of light path (cm)

c = concentration (mol/L)

An ε value of 0.036 L mg^{-1} cm^{-1} was adopted from Demirer et al. (2019), and the path length was 1 cm. So, the concentration of the 10-fold diluted suspension was estimated to be 21.69 mg/L. The concertation of the original supernatant solution was 216.9 mg/L (**Table S1**). The SWCNT-COOH suspension was stored in 4°C refrigerator until next step.

3.2 Activation of SWCNT-COOH suspension

A 500 mM MES buffer (pH 4.5-5.0) was prepared using nuclease-free water. Three volumes of SWCNT-COOH suspension were added to appropriate volumes of 500 mM MES buffer to obtain different SWCNT-COOH concentrations (66, 80, 138 mg/L) in 100 mM MES buffer. For example, 9.22 ml of SWCNT-COOH suspension (216.9 mg/L) was mixed with 10.78 ml of nuclease free water to obtain 100 mg/L, which

was then added to 5 ml of 500 mM MES. The final solution concentration was 80 mg/L. pH was re-measured to ensure it was between 5-6, as EDC-NHS activation was optimal at pH between 4.5 and 6.

To activate the SWCNT-COOH suspension, a fresh EDC-NHS solution was prepared by adding 10 mg EDC and 10 mg NHS to 2.5 ml of 100 mM MES buffer, which was vortexed until completely dissolved, and then the solution was added dropwise (using a 1-ml syringe with an 18-gauge needle) to the SWCNT-COOH suspension. This solution was batch sonicated for 15 minutes at room temperature, followed by stirring on a stir plate at 500 rpm for 45 minutes. After the reaction, the activated SWCNT-COOH solution was split into two pre-washed 100,000-MWCO filter units. Up to 50 ml of 0.1× PBS buffer (pH 7.4) was added to each filter, followed by centrifugation at 500 g for 10 minutes. The flow-through was discarded. The wash step was repeated two more times to remove free EDC, NHS, and by-products. After the last wash step, the top parts of the filter unit were bath-sonicated (2~3 minutes) to recover the activated COOH-SWNTs. A typical recovery volume from each filter was around 4~5 mL. All the activated SWNT-COOH solutions were combined in one tube after recovery from filters. To this combined solution, 0.1× PBS (pH 7.4) buffer was added until reaching the initial COOH-SWCNT solution volume. Finally, the activated COOH-SWCNT solution was re-suspended via bath sonication for 15 minutes.

3.3 PEI functionalization, washing and re-suspension of the PEI-SWCNT product

25-kDa bPEI (40-60 mg) was added to 5 ml of 0.1× PBS buffer and was dissolved completely by vortexing (5-10 minutes) and bath sonication (2-3 minutes); pH was adjusted to 7.4-7.6 using 5M HCl. The above activated (coupling reagent grafted) COOH-SWCNT was added to the PEI solution dropwise. The solution-containing vial was placed on an orbital shaker set at 180 rpm to allow overnight reaction (16 hours). The amination reaction was most efficient at pH 7-8 so the pH was adjusted if needed. The PEI-SWCNT reaction solution was washes six times with nuclease-free water using the 100,000-MWCO filters. Centrifugation speeds (around 500g) were adjusted to avoid agglomeration. Flow-through was discarded. Finally, PEI-SWCNT was added into MES buffer and resuspension was achieved by probe-tip sonication for 10 minutes in an ice bath. (Probe tip condition: 10% amplitude, pulse ON for 1 sec and pulse OFF for 2 sec for 5 minutes, and a final 5-minute continuous sonication).

The re-suspended PEI-SWCNT solution was centrifuged at 16,000g for 1 hour at room temperature to obtain the supernatant. Absorbance was measured as described in the "*Preparation of SWCNT-COOH suspension*" section, and synthesis efficiency was calculated as the ratio of obtained PEI-SWCNT to added COOH-SWCNT (see equation below and **Table S1**). In addition, zeta potential, particle size distribution and polydispersity index (PDI) were measured using Malvern Zetasizer Nano ZS to assess the colloidal stability and confirm PEI attachment (**Table S2** and **Figures S18** to **S25**). Batch 1A of the prepared PEI-SWCNT was used for toxicity testing.

$$Efficiency = \frac{Mass\ of\ PEI - SWCNTs\ obtained\ in\ final\ step}{Mass\ of\ COOH - SWCNTs\ added\ to\ the\ reaction}\ x\ 100\%$$

Table S1. Absorbance, concentration and efficiency of three PEI-SWCNT batches

Sample	Abs @ 632 nm for 10x diluted	Concentration for 10x diluted	Concentration of the original solution	Efficiency *
COOH-SWCNT	0.781	21.69 mg/L	216.9 mg/L	N/A
PEI-SWCNT (1A)	0.497	13.80 mg/L	138.0 mg/L	63.64%
PEI-SWCNT (1B)-diluted to 100 mg/L	0.290	8.055 mg/L	80.55 mg/L	80.55%
PEI-SWCNT (1C)-diluted to 100 mg/L	0.239	6.638 mg/L	66.38 mg/L	66.38%

* Efficiency (%) was calculated using the above equation. 1A, 1B and 1C refer to batch numbers.

Table S2. Characterization of COOH- and bPEI-functionalized SWCNT samples using a Zetasizer. Characteristic parameters include average Zeta potential, Z-average (intensity weighted harmonic mean of particle size distribution), and PDI (polydispersity index). See **Figures S17 to S24** for more info.

**Sample**	**Average zeta potential**	**Z-average (nm)**	**PDI**
COOH-SWCNT	-57.3 ± 12.0	157.3	0.284
PEI-SWCNT (1A)	+62.7 ± 7.56	152.7	0.375
PEI-SWCNT (1B)-diluted to 100 mg/L	+61.5 ± 7.22	144.9	0.280
PEI-SWCNT (1C)-diluted to 100 mg/L	+64.1 ± 8.38	169.2	0.281

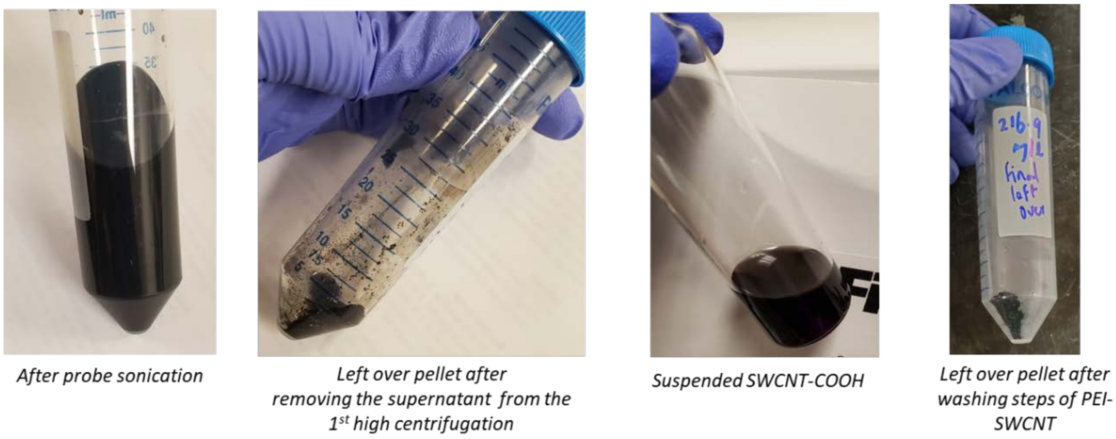

Figure S17. Physical appearance of SWCNT-COOH and PEI-SWCNT

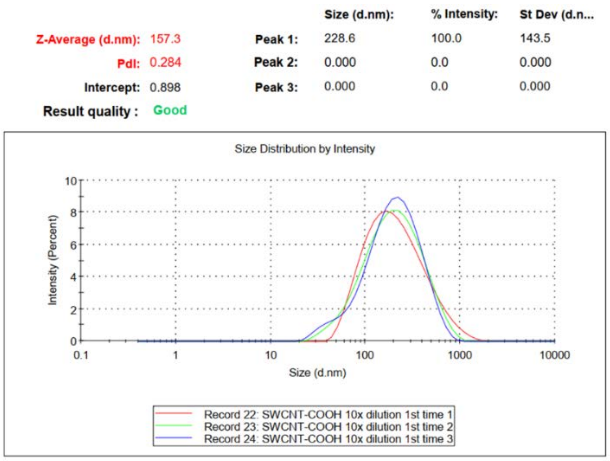

Figure S18. Particle size distribution for non-functionalized SWCNT-COOH

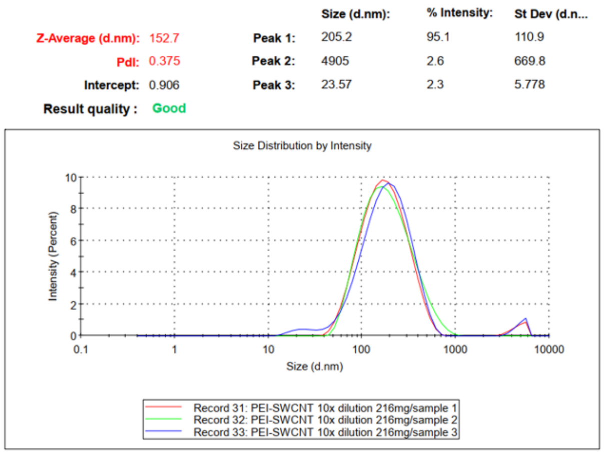

Figure S19. Particle size distribution for PEI-SWCNT (Batch 1A)

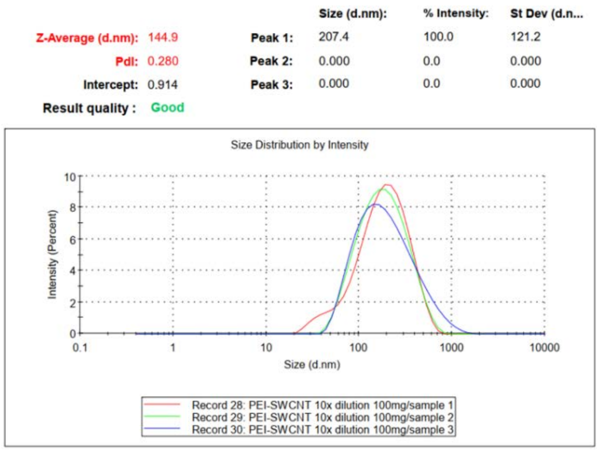

Figure S20. Particle size distribution for PEI-SWCNT (Batch 1B)

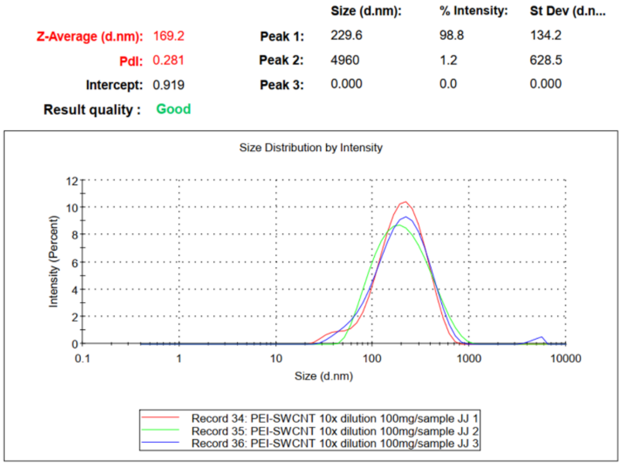

Figure S21. Particle size distribution for PEI-SWCNT (Batch 1C)

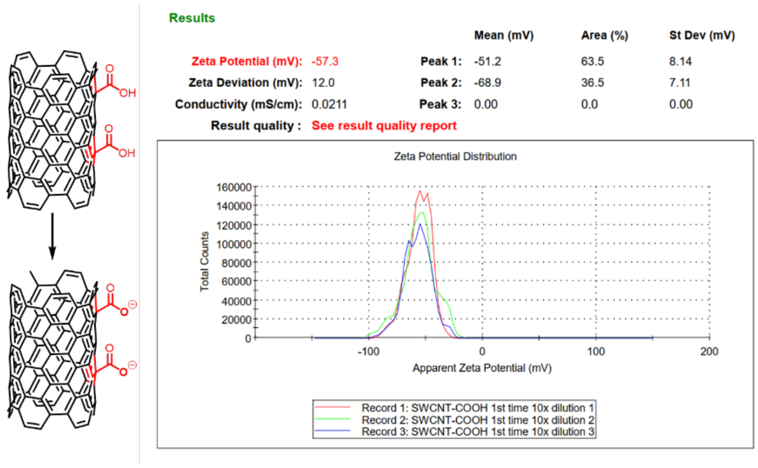

Figure S22. Zeta potential for SWCNT-COOH

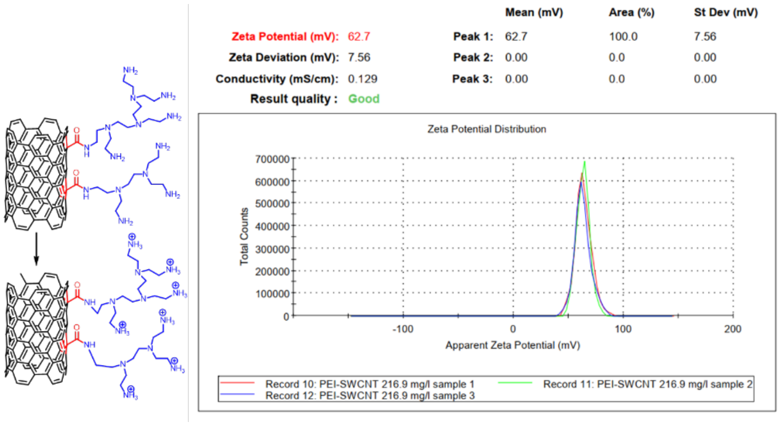

Figure S23. Zeta potential for PEI-SWCNT (Batch 1A)

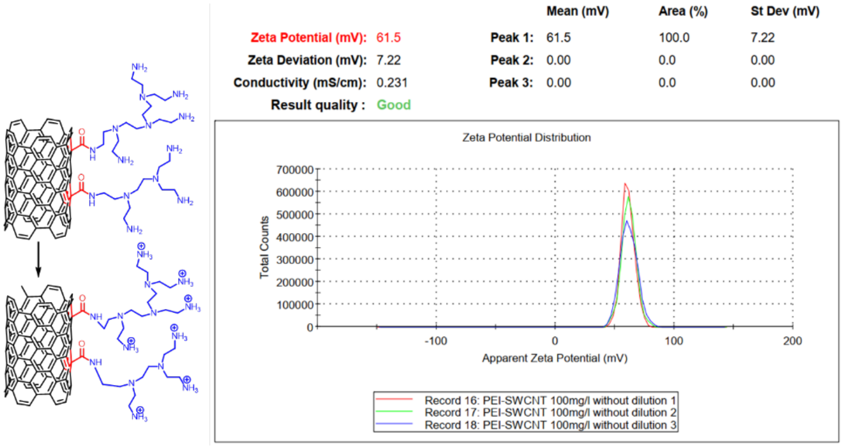

Figure S24. Zeta potential for PEI-SWCNT (Batch 1B)

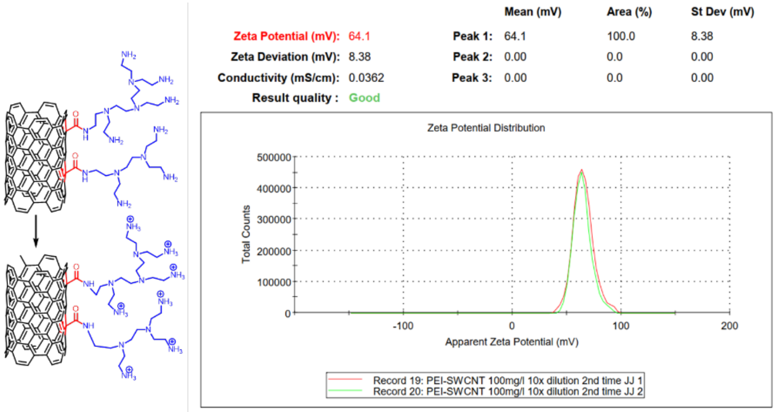

Figure S25. Zeta potential for PEI-SWCNT (Batch 1C)

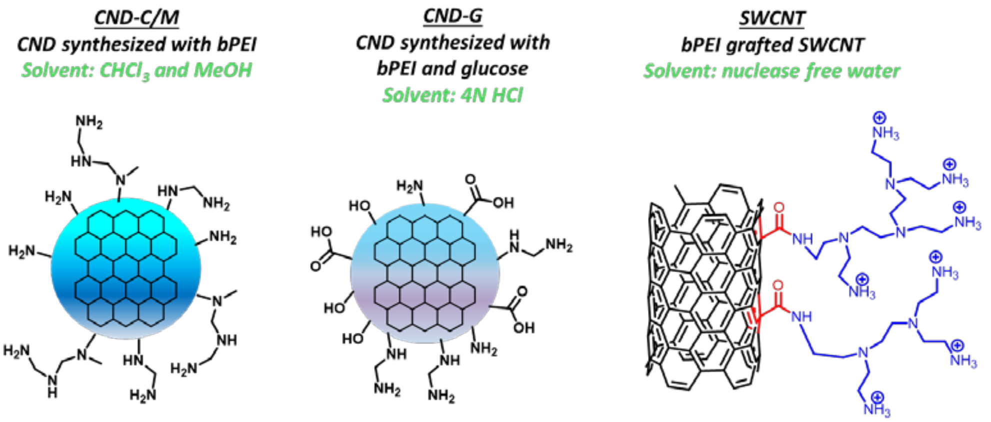

Figure S26. Molecular structures of three non-metallic nanoparticle particles (NMNPs) designated as CND-C/M, CND-G, and SWCNT

Part 4. Effects of 48-hr exposure to five nanoparticles (CND-G, CND-C/M, CND-G-A14, CPP, and SWCNT) at two dosages (high and low) on nine cyanobacterial strains. Effects are expressed as percent change in treated samples over the control: (treated – control)/control × 100. Negative values indicate inhibition whereas positive values indicate stimulation. These results are presented as Figures 3-7 in the main body of published article.

Table S3. Effects of 48-hr exposure to five NPs on nine cyanobacterial strains measured as OD_{750} (mean/standard deviation of percent change over the control, n = 3). Statistical significance level: * $P < 0.05$, ** $P < 0.01$, *** $P < 0.001$ (ANOVA with the post-hoc Dunnett test)

**Pure culture cyanobacterial strain**	CND-G_High	CND-G_Low	CND-C/M_High	CND-C/M_Low	CND-G-A14_ High	CND-G_A14 _Low	γ-Zein-CADY _High	γ-Zein-CADY _Low	SWCNT _High	SWCNT _Low
Microcoleus autumnale CAWBG635	3/17.4	0/8.4	11/13.9	9/0.8	329/88.2 ***	-5/95.4	29/19.7 **	0/14.1	14/58.0	-22/8.9
Lyngbya sp. CCAP 1446/10	-41/157	-67/ 36.3	-2/132	-26/73.6	-47/34.5	-59/ 55.8	-17/80.2	-27/85.0	8/34.3	-27/34.0
Planktothrix agardhii SB 1810	-43/2.7 ***	1/5.0	-38/9.3 **	-7/7.6	-70/3.3 ***	-76/5.1 ***	-27/8.1 ***	-2/3.3	-47/3.2 ***	-61/6.4 ***
Anabaena cylindrica PCC 7122	-4/4.6	-2/8.4	-2/9.7	8/0.4	-70/12.5 ***	-35/ 12.5 *	-6/9.1	5/10.1	-68/5.0 ***	-63/8.7 ***
Aphanizomenon sp. NZ	-10/3.5 **	-2/4.1	-12/9.5 *	0/2.5	-39/10.8 **	-30/ 14.4 *	-6/1.4 *	2/5.0	-42/2.7 ***	-54/4.1 ***
Microcystis aeruginosa LE3	-11/3.1	-1/11.0	-17/14.0	3/12.6	-38/7.4 **	-24/ 11.5 *	-80/7.9 **	-17/27.5	-73/27.6 **	-95/2.1 **
M. aeruginosa UTEX 2386	-7/8.1	4/5.2	-3/6.9	1/5.8	-19/8.9 **	-16/2.6 *	-7/4.7	-2/6.2	3/107.7	-56/2.5
M. aeruginosa UTEX 2385	-6/3.5	-1/5.1	-13/16.3	1/2.6	-45/8.9 **	-10/2.6 *	-5/3.5 *	3/2.6	-43/26.0	-47/23.4 *
Synechocystis sp. PCC 6803	-74/5.6 ***	-52/4.4 ***	-66/12.5 ***	-29/3.0 **	-75/5.1 ***	-70/8.4 ***	-76/4.9 ***	-71/14.4 ***	-31/1.2 **	-41/5.3 ***

Table S4. Effects of 48-hr exposure to five NPs on nine cyanobacterial strains measured as OD_{680} (mean/standard deviation of percent change over the control, n = 3). Statistical significance level: * P < 0.05, ** P < 0.01, *** P < 0.001 (ANOVA with the post-hoc Dunnett test)

**Pure culture cyanobacterial strain**	CND-G_High	CND-G_Low	CND-C/M_High	CND-C/M_Low	CND-G-A14_High	CND-G-A14_Low	γ-Zein-CADY_High	γ-Zein-CADY_Low	SWCNT_High	SWCNT_Low
Microcoleus autumnale CAWBG635	-3/21.6	-5/13.9	4/17.9	4/4.3	600/459	-34/110	24/17.3	-5/21.0	10/81.9	-30/11.0
Lyngbya sp. CCAP 1446/10	-46/166	-70/38.0	-11/116	-32/72.9	-49/34.8	-61/57.6	-24/86.3	-34/88.0	-13/26.2	-43/27.1 *
Planktothrix agardhii SB 1810	-37/3.7 ***	1/5.0	-32/8.8 **	-7/6.5	-57/4.5 ***	-66/7.7 ***	-23/8.1 **	-2/2.5	-43/4.5 ***	-57/7.0 ***
Anabaena cylindrica PCC 7122	-6/5.5	-7/9.9	-5/12.2	6/0.3	-67/11.9 **	-33/12.6 *	-11/10.6	3/14.5	-71/3.6 ***	-69/11.2 ***
Aphanizomenon sp. NZ	-9/1.0 ***	0/2.8	-10/8.5	2/1.5	-39/8.2 **	-30/12.0 **	-5/1.4 *	2/3.1	-44/2.0 ***	-54/3.8 ***
Microcystis aeruginosa LE3	-18/4.8 **	-3/8.5	-20/13.9 *	0/9.2	-37/6.8 **	-21/12.0 *	-82/20.1 **	-20/6.4	-76/3.2 **	-95/4.3 ***
M. aeruginosa UTEX 2386	-8/9.2	3/6.8	-4/8.3	1/7.0	-18/9.3 *	-16/2.8 *	-5/5.2	-2/6.9	-53/9.1 ***	-55/1.8 ***
M. aeruginosa UTEX 2385	-7/3.6	-1/5.3	-14/16.4	1/2.2	-45/9.3 *	-10/2.8 *	-6/3.5 **	1/3.0	-51/11.1 **	-53/9.4 **
Synechocystis sp. PCC 6803	-76/5.2 ***	-52/4.2 ***	-67/12.7 ***	-28/3.8 **	-77/5.3 ***	-72/8.1 ***	-77/5.0 ***	-72/14.6 ***	-36/0.8 **	-45/5.8 ***

Table S5. Effects of 48-hr exposure to five NPs on nine cyanobacterial strains measured as FE_{450} (mean/standard deviation of percent change over the control, n = 3). Statistical significance level: * P < 0.05, ** P < 0.01, *** P < 0.001 (ANOVA with the post-hoc Dunnett test)

**Pure culture cyanobacterial strain**	CND-G_High	CND-G_Low	CND-C/M_High	CND-C/M_Low	CND-G-A14_High	CND-G-A14_Low	γ-Zein-CADY_High	γ-Zein-CADY_Low	SWCNT_High	SWCNT_Low
Microcoleus autumnale CAWBG635	19/27.9	5/20.2	87/84.6	-31/36.2	338/355	-27/97.0	46/45.0	-18/29.9	-18/84.5	-62/14.4
Lyngbya sp. CCAP 1446/10	-16/275	-68/39.6	-19/90.2	-33/93.0	-68/46.3	-55/91.0	-12/153	-35/118	-56/24.8	-82/10.9 *
Planktothrix agardhii SB 1810	-28/11.3 *	15/21.8	-19/20.7	2/7.1	-43/8.9 **	-64/8.4 ***	8/20.3	-4/7.2	-35/3.2 ***	-45/7.9 ***
Anabaena cylindrica PCC 7122	-11/10.4	-14/4.4	-14/13.6	-12/12.2	-54/9.8 **	-37/5.6 **	-17/6.4	-3/9.4	-77/0.7 ***	-80/3.7 ***
Aphanizomenon sp. NZ	-12/8.1	2/5.3	-9/10.2	-1/7.2	-43/4.4	-64/13.5	-6/4.7	-1/1.5	-29/4.8 ***	-35/5.9 ***
Microcystis aeruginosa LE3	-24/7.5 **	-3/8.6	-21/8.9	-3/11.6	-22/4.4 **	-13/3.4 *	-61/7.6 **	-24/25.3	-50/30.0 *	-79/2.6 **
M. aeruginosa UTEX 2386	-19/10.0 *	-10/5.4	-11/10.3	-15/9.2	-4/14.3	-9/5.7	-10/8.6	-9/3.1	-59/4.6 ***	-49/5.5 ***
M. aeruginosa UTEX 2385	-6/7.7	-3/11.2	-14/7.4	-3/4.0	-32/22.5 *	4/22.4	-5/5.3	-6/3.2	-55/7.5 **	-43/12.0 **
Synechocystis sp. PCC 6803	-56/12.7 ***	-28/15.2 *	-43/26.4 *	-14/10.4	-72/9.4 **	-75/10.7 **	-56/12.5 *	-54/20.0 *	-15/8.8	-17/7.2

Table S6. Effects of 48-hr exposure to five NPs on nine cyanobacterial strains measured as FE_{620} (mean/standard deviation of percent change over the control, n = 3). Statistical significance level: * P < 0.05, ** P < 0.01, *** P < 0.001 (ANOVA with the post-hoc Dunnett test)

**Pure culture cyanobacterial strain**	CND-G_High	CND-G_Low	CND-C/M_High	CND-C/M_Low	CND-G-A14_High	CND-G-A14_Low	CPP_High	CPP_Low	SWCNT_High	SWCNT_Low
Microcoleus autumnale CAWBG635	40/90.3	-5/46.3	194/123	-21/41.8	294/380	-23/78.9	44/19.0	-34/45.4	-19/102	-53/14.5
Lyngbya sp. CCAP 1446/10	-28/310	-76/33.2	-22/94.9	-33/102	-71/41.1	-61/134	-13/207	-40/155	-57/32.6	-85/10.1 *
Planktothrix agardhii SB 1810	-39/3.7 ***	15/6.4 *	-35/18.8 *	8/4.1	-17/25.4	-89/3.3 ***	3/11.0	7/11.9	30/6.8 *	20/16.5
Anabaena cylindrica PCC 7122	-2/16.1	-7/18.7	0/22.5	14/3.0	33/33.9	-17/11.9	-3/14.2	13/21.7	-87/1.1 ***	-79/5.8 ***
Aphanizomenon sp. NZ	-9/12.3	-9/6.1	-15/10.1	-2/10.3	79/40.0	96/89.5	-23/4.6 **	-5/14.8	24/10.6 *	16/7.4
Microcystis aeruginosa LE3	-2/18.9	-2/1.3	-9/0.9	-1/4.9	-9/5.7	-2/11.9	75/39.0 *	-25/29.3	-37/12.5 ***	-55/1.0 ***
M. aeruginosa UTEX 2386	-3/12.7	11/2.9	7/7.3	9/9.2	-15/6.5 **	-14/1.5 **	3/13.0	0/10.9	-40/6.0 ***	-40/10.6 ***
M. aeruginosa UTEX 2385	-8/6.0	-4/12.3	-12/18.5	0/1.6	-25/15	14/22	-3/7.1	2/4.8	-51/33.7 *	-27/11.9
Synechocystis sp. PCC 6803	-50/1.1 ***	-17/4.6 *	-34/12.6 *	3/11.6	-70/9.4 ***	-66/7.1 **	-49/7.8 *	-46/28.8 *	-94/71.9	108/28.0

Table S7. Effects of 48-hr exposure to five NPs on nine cyanobacterial strains measured as QY (mean/standard deviation of percent change over the control, n = 3). Statistical significance level: * P < 0.05, ** P < 0.01, *** P < 0.001 (ANOVA with the post-hoc Dunnett test)

**Pure culture cyanobacterial strain**	CND-G_High	CND-G_Low	CND-C/M_High	CND-C/M_Low	CND-G-A14_High	CND-G- A14_Low	γ-Zein-CADY_High	γ-Zein-CADY_Low	SWCNT_High	SWCNT_Low
Microcoleus autumnale CAWBG635	1349/NA	3948/NA *	2198/NA	900/NA	153/62.1	-4/141	2899/NA	-100/ NA	-27/111	-86/24.9
Lyngbya sp. CCAP 1446/10	16/39.4	27/29.3	47/67.1	45/43.5	-29/26.6	-26/ 51.3	24/27.1	47/28.0	-51/17.2 **	-57/8.6 **
Planktothrix agardhii SB 1810	-12/15.5	-8/3.1	-5/22.3	-5/7.4	-98/0.4 ***	-98/0.4 ***	3/12.0	-2/5.2	-98/2.6 ***	-97/4.9 ***
Anabaena cylindrica PCC 7122	-6/10.2	-7/9.2	-7/28.9	9/8.2	-43/57.3	27/52.1	-8/20.1	-7/6.4	-98/3.2 ***	-96/4.3 ***
Aphanizomenon sp. NZ	-2/13.3	6/7.4	3/5.1	-6/6.6	-66/18.9 **	-22/ 17.9	-5/12.1	-8/5.7	-99/1.1 ***	-99/0.9 ***
Microcystis aeruginosa LE3	-13/17.2	-5/12.1	-9/14.1	-5/11.0	-18/2.1	-12/ 17.7	-100/0 ***	-4/11.1	-91/4.9 *	-96/25.4 *
M. aeruginosa UTEX 2386	-1/2.9	1/9.3	4/6.2	2/0.0003	-9/7.5	1/13.4	3/1.1	-6/5.9	-97/4.4 ***	-99/2.3 ***
M. aeruginosa UTEX 2385	-11/45.2	-23/19.8	-7/19.8	-23/13.8	13/28	11/28	-5/40.8	-15/17.4	-98/3.6 **	-91/14.6 **
Synechocystis sp. PCC 6803	-15/24.5	-5/17.0	-17/33.9	9/8.6	7/16.6	-8/11.2	-13/20.2	-14/10.8	-88/10.8 ***	-87/11.4 ***

Table S8. Number of statistically significant treatments (SSTs) as evaluated by five measurement endpoints for each of the nine tested cyanobacterial strains. Total number of treatments per strain and endpoint = 2 doses (high + low) × 5 NPs (CND-G + CND-C/M + CND-G-A14 + CPP + SWCNT) = 10. The SSTs are broken down by significance level (i.e., P<0.05, P<0.01, and P<0.001). The number of significant stimulative treatment is shown in parenthesis.

	OD750				OD680				FE450				FE620				QY			
Genus or Strain	SST	P<0.05	P<0.01	P<0.001	SST	P<0.05	P<0.01	P<0.001	SST	P<0.05	P<0.01	P<0.001	SST	P<0.05	P<0.01	P<0.001	SST	P<0.05	P<0.01	P<0.001
Microcoleus	2	0	(1)	(1)	0	0	0	0	0	0	0	0	0	0	0	0	1	(1)	0	0
Lyngbya	0	0	0	0	1	1	0	0	1	1	0	0	1	1	0	0	2	0	2	0
Planktothrix	7	0	1	6	7	0	2	5	5	1	1	3	5	3(2)	0	2	4	0	0	4
Anabaena	4	1	0	3	4	1	1	2	4	0	2	2	2	0	0	2	2	0	0	2
Aphanizomenon	7	3	2	2	6	1	2	3	2	0	0	2	2	(1)	1	0	3	0	1	2
Ma LE3	5	1	4	0	7	2	4	1	6	2	4	0	3	(1)	0	2	3	2	0	1
Ma UTEX2386	2	1	1	0	4	2	0	2	3	1	0	2	4	0	2	2	2	0	0	2
Ma UTEX2385	4	3	1	0	5	2	3	0	3	1	2	0	1	1	0	0	2	0	2	0
Synechocystis	10	0	2	8	10	0	2	8	7	4	2	1	7	4	1	2	2	0	0	2
All 9 strains	41	9	12	20	44	9	14	21	31	10	11	10	25	11	4	10	21	3	5	13

Table S9. Number of statistically significant treatments (SSTs) in nine tested cyanobacterial strains as evaluated by five measurement endpoints for each of the five NPs. Total number of treatments per NP and endpoint = 2 doses (high + low) × 9 strains = 18. The SSTs are broken down by significance level (i.e., P<0.05, P<0.01, and P<0.001). The number of significant stimulative treatment is shown in parenthesis.

	OD750				OD680				FE450				FE620				QY			
NP	SST	P<0.05	P<0.01	P<0.001	SST	P<0.05	P<0.01	P<0.001	SST	P<0.05	P<0.01	P<0.001	SST	P<0.05	P<0.01	P<0.001	SST	P<0.05	P<0.01	P<0.001
SWCNT	11	1	3	7	15	1	4	10	13	2	3	8	10	4(2)	0	6	16	2	4	10
CND-G-A14	15	5	4	6(1)	14	6	4	4	9	2	6	1	5	0	3	2	3	0	1	2
CND-G	4	0	1	3	5	0	1	4	5	3	1	1	4	2(1)	0	2	1	(1)	0	0
CND-C/M	4	1	2	1	4	1	2	1	1	1	0	0	2	2	0	0	0	0	0	0
CPP	7	2	2(1)	3	6	1	3	2	3	2	1	0	4	3(1)	1	0	1	0	0	1
All 5 NPs	41	9	12	20	44	9	14	21	31	10	11	10	25	11	4	10	21	3	5	13

Table S10. Average degree of alteration for the five measurement endpoints by NP treatments in the nine tested cyanobacterial strains (negative values indicating inhibitory effects whereas positive values representing stimulative effects)

	OD750					OD680					FE450					FE620					QY				
Genus or Strain	SWCNT	A14	CND-G	CND-C/M	CPP	SWCNT	A14	CND-G	CND-C/M	CPP	SWCNT	A14	CND-G	CND-C/M	CPP	SWCNT	A14	CND-G	CND-C/M	CPP	SWCNT	A14	CND-G	CND-C/M	CPP
Microcoleus	-4	162	1.5	10	14.5	-10	283	-4	4	9.5	-40	155.5	12	28	14	-36	135.5	17.5	86.5	5	-56.5	74.5	2648.5	1549	1399.5
Lyngbya	-9.5	-53	-54	-14	-22	-28	-55	-58	-21.5	-29	-69	-61.5	-42	-26	-23.5	-71	-66	-52	-27.5	-26.5	-54	-27.5	21.5	46	35.5
Planktothrix	-54	-73	-21	-22.5	-14.5	-50	-61.5	-18	-19.5	-12.5	-40	-53.5	-6.5	-8.5	2	25	-53	-12	-13.5	5	-97.5	-98	-10	-5	0.5
Anabaena	-65.5	-52.5	-3	3	-0.5	-70	-50	-6.5	0.5	-4	-78.5	-45.5	-12.5	-13	-10	-83	8	-4.5	7	5	-97	-8	-6.5	1	-7.5
Aphanizomenon	-48	-34.5	-6	-6	-2	-49	-34.5	-4.5	-4	-1.5	-32	-53.5	-5	-5	-3.5	20	87.5	-9	-8.5	-14	-99	-44	2	-1.5	-6.5
Ma LE3	-84	-31	-6	-7	-48.5	-85.5	-29	-10.5	-10	-51	-64.5	-17.5	-13.5	-12	-42.5	-46	-5.5	-2	-5	25	-93.5	-15	-9	-7	-52
Ma UTEX2386	-26.5	-17.5	-1.5	-1	-4.5	-54	-17	-2.5	-1.5	-3.5	-54	-6.5	-14.5	-13	-9.5	-40	-14.5	4	8	1.5	-98	-4	0	3	-1.5
Ma UTEX2385	-45	-27.5	-3.5	-6	-1	-52	-27.5	-4	-6.5	-2.5	-49	-14	-4.5	-8.5	-5.5	-39	-5.5	-6	-6	-0.5	-94.5	12	-17	-15	-10
Synechocystis	-36	-72.5	-63	-47.5	-73.5	-40.5	-74.5	-64	-47.5	-74.5	-16	-73.5	-42	-28.5	-55	7	-68	-33.5	-15.5	-47.5	-87.5	-0.5	-10	-4	-13.5
Average-all strains	-41	-22	-17	-10	-17	-49	-7	-19	-12	-19	-49	-19	-14	-10	-15	-29	2	-11	3	-5	-86	-12	291	174	149
